# Differences in the clinical characteristics of papillary thyroid microcarcinoma located in the isthmus ≤5 mm and >5mm in diameter

**DOI:** 10.3389/fonc.2022.923266

**Published:** 2022-08-01

**Authors:** Feng Zhu, Lixian Zhu, Yibin Shen, Fuqiang Li, Xiaojun Xie, Yijun Wu

**Affiliations:** The Department of Thyroid Surgery, The First Affiliated Hospital, School of Medicine, Zhejiang University, Hangzhou, China

**Keywords:** papillary thyroid microcarcinoma, isthmus, tumor size, clinicopathological features, recurrence

## Abstract

**Background:**

The optimal treatment of papillary thyroid microcarcinomas (PTMCs) located in the isthmus (iPTMCs) is still controversial. The purpose of this study was to compare the clinicopathologic features of patients with iPTMCs ≤5 mm and >5 mm in diameter after total thyroidectomy, and to identify the risk factors for recurrence in patients with iPTMCs.

**Methods:**

A total of 102 iPTMC patients who underwent total thyroidectomy were reviewed retrospectively. The clinicopathologic characteristics of iPTMCs ≤5 mm group (*n* = 29) have been compared with a group >5 mm (*n* = 73). Univariate and multivariate Cox proportional hazard models served to identify risk factors associated with recurrence-free survival (RFS).

**Results:**

Gender (*p* = 0.033), multifocality (*p* = 0.041), and central lymph node metastasis (CLNM) (*p* = 0.009) of patients in the ≤5 mm and >5 mm groups differed significantly. iPTMC patients with age <55 years, male, multiple tumor, and extrathyroidal extension showed comparatively more frequent of CLNM in >5 mm groups. Of the 102 patients, nine (8.8%) developed recurrence during follow-up (median: 49.5 months). The patients with recurrences had comparatively high rates of CLNM (*p* = 0.038), extranodal invasion (*p* = 0.018), and more MNCND (Metastasis Nodes for Central Neck dissection) (*p* = 0.020). A cutoff of MNCND >2.46 was established as the most sensitive and specific level for the prediction of recurrence based on receiver operating characteristic (ROC) curve analyses. Multivariate analysis showed that the number of MNCND ≥3 was an independent predictor of poor RFS (*p* = 0.028).

**Conclusion:**

We have found that the recurrence rates are similar in patients with iPTMCs ≤5 mm and >5 mm. The iPTMCs >5 mm were more likely to be associated with pathological features such as multifocality and CLNM. The male gender, extrathyroidal extension, and CLNM were associated with recurrence of iPTMCs except for tumor size and multifocality. Higher risk of CLNM should be considered in iPTMC >5 mm when it reaches some risk factors. The numbers of MNCND ≥3 may be an independent predictor for recurrence, which could help clinicians for the decision of radioiodine administration and the modulation of follow-up modalities.

## Introduction

Papillary thyroid microcarcinoma (PTMC) is papillary thyroid carcinoma (PTC) with a diameter less than 10 mm. The incidence of PTC has increased rapidly worldwide in recent years (1). About half of the increase being because of PTMC, which has increased more than fourfold over the past 30 years (2, 3). The incidence of PTC arising in the isthmus was reported to be between 2.2% and 12.3% (4, 5). Compared with PTC in other sites, iPTC tended to be smaller in size and had a higher proportion of PTMC.

Until now, the clinical significance of iPTMCs and their optimal surgical strategy remains controversial. The American Thyroid Association (ATA) guidelines include PTMCs of absence of aggressive features in the low-risk category. Previous studies have demonstrated that PTMCs in the isthmus show more aggressive behavior and are more likely to be associated with multifocal disease, lymph node involvement, and capsule invasion than in other thyroid regions (6). Total thyroidectomy is to be considered as an appropriate surgical treatment when the iPTMCs patient falls within the high-risk category (7). However, the patient may be suitable for thyroid isthmusectomy if the iPTMCs are small in size and have no aggressive characteristics (8).

The diagnosis of iPTMC ≤5 mm has increased with the development of diagnostic technology. Meanwhile, the clinical significance of iPTMCs ≤5 mm and their optimal management remain unclear. Some studies have demonstrated that PTMCs ≤5 mm and >5mm in diameter have also been suggested as being important for risk stratification. PTMC >5mm was more likely to have high-risk features (9–12). To our knowledge, the clinicopathologic features and extent of surgery of iPTMCs ≤5 mm and >5 mm remain undetermined. The objective of this study was to evaluate the clinicopathologic characteristics and recurrence rates in patients with iPTMCs ≤5 mm and >5 mm who underwent total thyroidectomy at our institution. Additionally, we sought to investigate the risk factors associated with CLNM and recurrence.

### Patients and methods

A total of 102 iPTMC patients (82 women and 20 men) who underwent total thyroidectomy and bilateral central neck dissection between January 2015 and January 2020 in the Department of Thyroid Surgery of the First Affiliated Hospital, School of Medicine, Zhejiang University were analyzed retrospectively. Before surgery, each patient underwent ultrasound-guided fine-needle aspiration biopsy (FNAB). iPTMC was defined as a PTC located in the isthmus less than 1 cm in size according to final pathologic reports. The clinicopathological data of iPTMC patients were analyzed. These patients were classified into two groups: iPTMC ≤5 mm (*n* = 29) and iPTMC >5 mm (*n* = 73). This retrospective study was approved by the Clinical Research Ethics Committee of the First Affiliated Hospital, School of Medicine, Zhejiang University. Written informed consent was obtained from all patients before the study.

The iPTMC patients in the study met the following inclusion criteria: the center of the tumor located between two imaginary lines perpendicular to the surface of the skin from the most lateral points of the trachea. Patients who underwent unilateral lobectomy or isthmectomy, secondary surgery, and other types of thyroid cancer were excluded. iPTMCs less than 3 mm were also excluded due to the difficulty of preoperative identification. The 8th edition of the American Joint Committee on cancer (AJCC), TNM classification of malignant tumors in 2016 was used to describe and categorize cancer stages. Radio-iodine therapy was not performed postoperatively. All patients received thyroid-stimulating hormone-suppressive therapy after surgery. Postsurgical physical examinations were performed every 3 to 6 months. The mean follow-up period was 45.2 ± 18.0 months (median: 49.5 months). Cervical CT and FNAB were performed during a follow-up to evaluate suspected recurrences. Reoperation was performed on patients’ suspected recurrence, and postoperative pathology was confirmed.

### Statistical analysis

Statistical analyses were performed using SPSS version 22.0 (SPSS, Chicago, IL). The results were expressed as mean ± SD. Differences between categorical variables were assessed using the Chi-square test or Fisher exact test and continuous variables using Student’s *t*-test. Multivariate recurrence analysis was conducted using the Cox proportional hazards regression to identify independent prognostic factors. The restricted mean survival time (RMST) was used for survival analysis of variables of CLMN, CLNM ratio, and MNCND, which were not suitable for log-rank test. The time for calculating the RMSTs was set at t* = 60 months. Statistical analysis for cutoff point was assessed by time-dependent ROC curves and was performed using R version 4.2.0 (R Core Team (2022), R Foundation for Statistical Computing, Vienna, Austria), applying the package time ROC for time-dependent ROC analysis. The optimal cutoff point was selected as the closest to (0.1) the criteria. RFS was conducted between the different groups using the Kaplan–Meier analysis, and the differences in curves for each variable were determined by the log-rank test. Results were reported as hazard ratios (HRs) and 95% confidence intervals (95% CI). Differences with *P* < 0.05 were defined as statistically significant.

## Results

This study included 102 patients of mean age 42.38 ± 11.38 years (range: 22–77 years). The clinicopathologic features of the two groups are shown in **Table 1**. There were no significant differences in age, thyroglobulin, and thyroidperoxidase antibodies between the ≤5 mm and >5 mm groups at the time of diagnosis. The mean size of the largest tumor was 4.5 ± 0.69 mm in the ≤5 mm group and 7.8 ± 1.43 mm in the >5 mm group. The rate of female patients in the two groups were 93.1% and 75.3%, respectively (*P* = 0.033). The rates of multifocality (five of 29 [17.2%] *vs*. 27 of 73 [37.0%]; *p* = 0.041) and CLNM (seven of 29 [24.1%] *vs*. 38 of 73 [52.1%]; *p* = 0.009) were significantly lower in the ≤5 mm than in the >5 mm group. However, there were no statistically significant differences in extrathyroidal extension, TNM stage, and recurrence (*p* = 0.442, *p* = 0.526, and *p* = 0.733, respectively). N1a and N1b classifications were observed in 24.1% and 0% in the ≤5 mm group and in 47.9% and 4.1% in the >5 mm group (*p* = 0.031).

**Table 1:** Clinical characteristics of patients with papillary thyroid microcarcinoma located in the isthmus by size.

Variables	≤5 mm group (*n=29*)	>5 mm group (*n* =73)	*p* value
Age (mean±SD, years)	45.0±11.5	41.4±11.3	0.150
<55	23 (79.3%)	63 (86.3%)	
≥55	6 (20.7%)	10 (13.7%)	0.277
No. of females, n (%)	27 (93.1%)	55 (75.3%)	0.033
Size of tumor, mm	4.5±0.69	7.8±1.43	—
Multifocality,n (%)	5 (17.2%)	27 (37.0%)	0.041
Extrathyroidal extension,n (%)	6 (20.7%)	18 (24.7%)	0.442
CLNM,n (%)	7 (24.1%)	38 (52.1%)	0.009
Mean no. dissected nodes for CND	6.11±4.23 (1-20)	7.96±5.58 (1-27)	0.118
Mean no. metastasis nodes for CND	0.32±0.61 (0-2)	1.9±3.0 (0-14)	0.006
N classification (N0/N1a/N1b)	22/7/0	35/35/3	0.031
TNM stage (I/II)	29/0	72/1	0.526
Recurrence,n (%)	3 (10.3%)	6 (8.2%)	0.733
TG( mean±SD, ng/mL)	24.5±43.4	37.6±94.7	0.254
TPOAb( mean±SD, IU/mL)	304.1±1233.3	339.9±878.8	0.996

CLNM, Central lymph node metastases; CND, Central neck dissection; TG, Thyroglobulin; TPOAb, Thyroid peroxidase antibody.

While CLNM was more frequent in patients with <55 years (58.7% *vs*. 10.0%, *P* = 0.004), male gender (77.8% *vs*. 43.6%, *P* = 0.011), multifocality tumor (66.7% *vs*. 43.5%, *P* = 0.047), and extrathyroidal extension (72.2% *vs*. 45.5%, *P* = 0.043) in >5 mm group, there were no statistically significant differences in ≤5 mm group (<55 *vs*. ≥55, *P* = 0.121; male *vs*. female, *P* = 0.376; single *vs*. multifocality tumor, *P* = 0.362; extrathyroidal extension present *vs*. absent, *P* = 0.554) (**Table 2**).

**Table 2:** Clinicopathologic factors related to central lymph node metastasis in >5 mm and ≤5 mm group.

Variables	≤5 mm group			>5 mm group		
	No. of patients (n = 29)	CLNM (n =7)	*p* value	No. of patients (n = 73)	CLNM (n =38)	*p* value
Age (years)						
<55	23	7(30.4%)	0.121	63	37(58.7%)	0.004
≥55	6	0(10.0%)		10	1(10.0%)	
Gender						
Male	2	1(50.0%)	0.376	18	14(77.8%)	0.011
Female	27	6(22.2%)		55	24(43.6%)	
Multifocality						
Single tumor	24	5(20.8%)	0.362	46	20(43.5%)	0.047
Multiple tumor	5	2(40.0%)		27	18(66.7%)	
Extrathyroidal extension						
Present	6	2(33.3%)	0.554	18	13(72.2%)	0.043
Absent	23	5(21.7%)		55	25(45.5%)	

CLNM, Central lymph node metastases.

During the follow-up period, nine (8.8%) patients developed recurrence. **Table 3** summarizes the nine patients who experienced tumor recurrence. The mean time to recurrence after surgery was 20.2 months (range: 2–50 months). Of these recurrences, the rate of CLNM, multifocality, and extrathyroidal extension was 88.9%, 44.4%, and 55.5%, respectively. All relapsed patients had lateral neck compartment metastases and seven (77.8%) were ipsilateral level III lymph node metastasis. Reoperative surgery was performed in patients with tumor recurrence.

**Table 3:** Characteristics of the patients with tumor recurrence.

Patient # no.	Age, y	Sex	Size, mm	CLNM(%)	Multifocality	Extrathyroidal extension	Time to disease free survival, m	Recurrence site
1	29	F	3	25%	**-**	**+**	2	Level VI lymph node
2	43	M	5	0%	**+**	**-**	50	Level III lymph node
3	34	F	3	25%	**+**	**-**	18	Level III lymph node
4	30	M	8	54%	**+**	**+**	17	Level II, III lymph node
5	32	M	7	100%	**-**	**-**	17	Level IV lymph node
6	51	F	10	63%	**-**	**+**	4	Level III lymph node
7	31	F	8	13%	**-**	**-**	32	Level III lymph node
8	38	M	7	13%	**+**	**+**	35	Level III, IV lymph node
9	43	F	6	43%	**-**	**+**	7	Level II, III lymph node

CLNM, Central lymph node metastases. + represents multifocal carcinoma or positive for Extrathyroidal extension. - Represents unifocal carcinoma or no Extrathyroidal extension.

**Figure 1:**
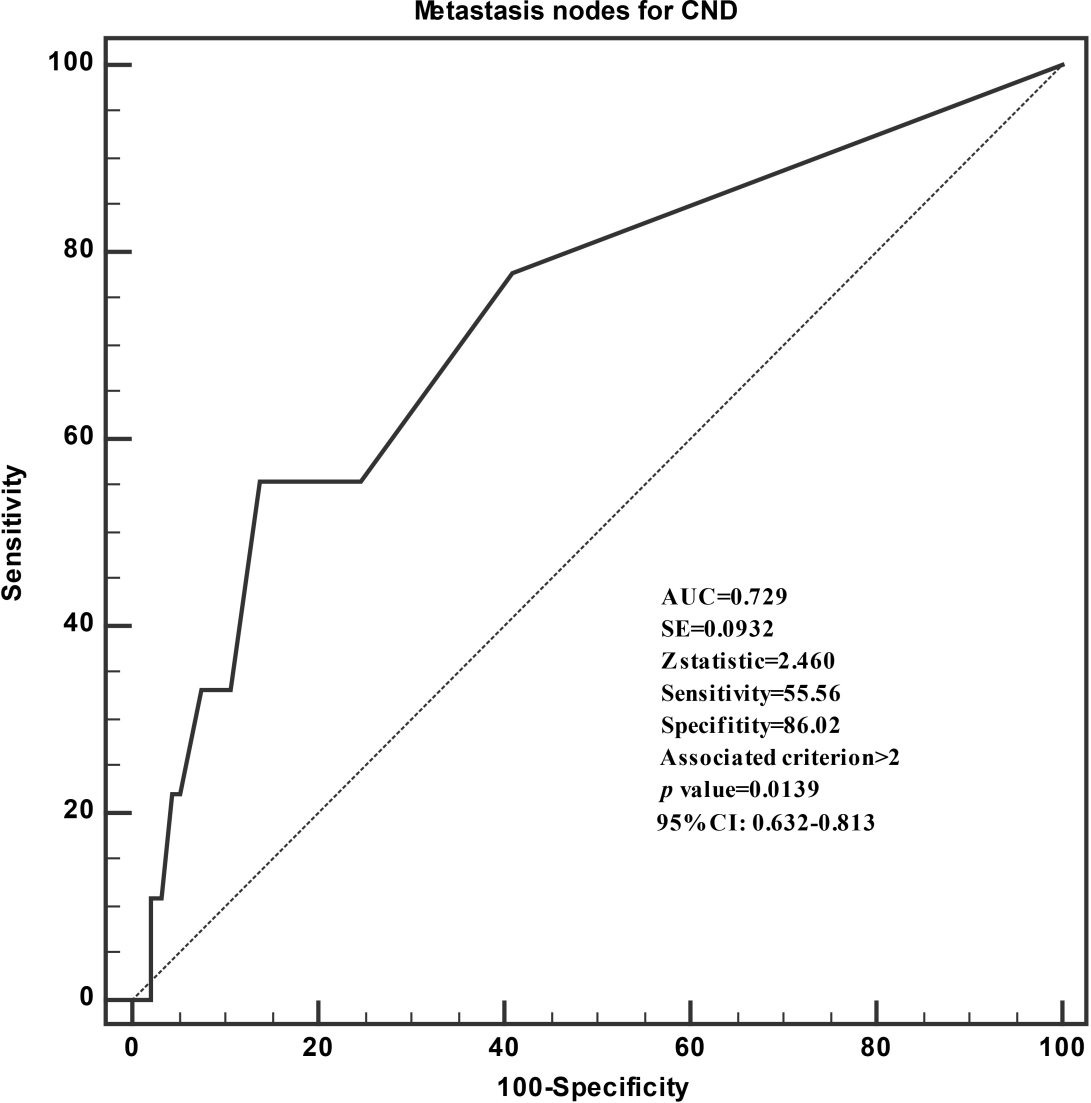
Receiver operating characteristic curve analyses of the numbers of lymph nodes metastases for predicting recurrence.

When the nine patients with recurrence were compared with the 93 patients without recurrence in terms of their clinicopathological variables, they were found to be more likely male (*p* = 0.049) and to have higher numbers of extrathyroidal extension (*p* = 0.018), higher numbers of CLNM (*p* = 0.038), and positive lymph nodes (*p* = 0.020; **Table 4**). ROC curve was performed to assess the recurrence based on the numbers of lymph nodes metastases (**Figure 1**). The area under the ROC curve (AUC) was 0.729 (*P* = 0.0139), which indicated that the numbers of lymph nodes metastases could accurately predict recurrence in iPTMCs. According to Youden’s index, the best cutoff value was 2.46. The sensitivity and specificity was 55.6% and 86.0%, respectively. Univariate analysis revealed that extrathyroidal extension and MNCND ≥3 associated significantly with poor RFS (*p* = 0.028 and *p* = 0.008, respectively). Multivariate analysis showed that MNCND ≥3 remained an independent variable for poor RFS (HR = 4.566 [95% CI 1.181–17.657]; *p* = 0.028) (**Table 5**).

**Table 4:** Clinicopathologic factors related to tumor recurrence.

Variables	Recurrence (n =9)	No recurrence (n = 93)	*p* value
Age (years), mean±SD	36.78±7.51	42.92±11.58	0.122
Age <55 years,n (%)	9 (100%)	77 (82.8%)	0.175
Male,n (%)	4 (44.4%)	16 (17.2%)	0.049
CLNM, n (%)	7 (77.8%)	38 (40.9%)	0.038
Multifocality, n (%)	4 (44.4%)	28 (30.1%)	0.376
Extrathyroidal extension, n (%)	5 (55.6%)	19 (20.4%)	0.018
Mean no. dissected nodes for CND	9.33±6.69	7.01±5.22	0.217
Mean no. metastasis nodes for CND	3.33±3.43	1.23±2.45	0.020
Ratio of CLNM	0.34±0.33	0.17±0.27	0.072

CLNM, Central lymph node metastases; CND, Central neck dissection.

**Table 5:** Univariate and Multivariate Analysis of Variables Associated with tumor recurrence.

Variables	Univariate		Multivariate	
	HR(95% CI)	*p* value	HR(95% CI)	*p* value
Sex, male	1.789 (0.927-3.452)	0.083		
Age, ≥55 years	0.038 (0.000-86.587)	0.407		
Tumor size, >5 mm	0.770 (0.192-3.082)	0.712		
CLNM	4.812 (0.996-23.258)	0.051		
Multifocality	1.608 (0.432-5.993)	0.479		
Extrathyroidal extension	4.351 (1.167-16.214)	0.028	3.173 (0.820-12.272)	0.094
≥3 metastasis nodes for CND	5.880 (1.578-21.913)	0.008	4.566 (1.181-17.657)	0.028

CLNM, Central lymph node metastases; CND, Central neck dissection.

**Figure 2:**
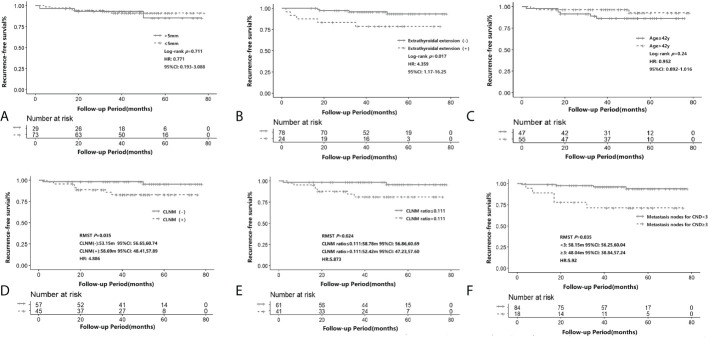
Recurrence-free survival according to tumor size (≤5 mm and >5mm) **(A)**, presence of extrathyroidal extension **(B)**, age (≤ 42y and >42y)**(C)**, CLNM **(D)**, CLNM ratio (≤0.111 and >0.111) **(E)** and the number of metastatic lymph nodes (> 2 and ≤2) **(F)** in iPTMC. The Kaplan–Meier method for recurrence with the log-rank test was used for statistical comparisons of **A-C**. The restricted mean survival time (RMST) was used for survival analysis of **D-F**. CLNM, central lymph node metastasis.

**Figure 2** shows the Kaplan–Meier estimates of RFS in iPTMC patients. Tumor size ≤5 mm patients or >5 mm patients did not have statistical differences for recurrence (*p* = 0.711, HR: 0.771, 95% CI: 0.193–3.088; **Figure 2A**). Compared with the extrathyroidal extension(-) group, the extrathyroidal extension(+) group had significantly lower RFS (*p* = 0.017, HR: 4.359, 95% CI: 1.17–16.25; **Figure 2B**). Time-dependent ROC analysis was conducted to clarify the best cutoff point of age and rate of CLNM. The cutoff points of age and CLNM ratio were 42 years and 0.111, respectively. CLNM(+) and CLNM ratio >0.111 was significantly related to the lower RFS (*p* = 0.035, RMST(CLNM[+]): 53.15m, 95% CI: 48.41–57.89, RMST(CLNM[-]): 58.69 m, 95% CI: 56.65–60.74, HR:4.886; *p* = 0.024, RMST(CLNM ratio >0.111): 52.42m, 95% CI: 47.23–57.60, RMST(CLNM ratio ≤0.111): 58.78m, 95% CI: 56.86–60.69, HR:5.873, respectively; **Figures 2D, E**). However, the cutoff points of age (>42 years and ≤42 years) did not have statistical differences for recurrence (*p* = 0.24; HR: 0.952, 95% CI: 0.892–1.016; **Figure 2C**). The RFS was significantly lower for the patients of metastasis nodes for CND ≥3 (*p* = 0.035, RMST (MNCND[+]): 48.04 m, 95% CI: 38.84–57.24, RMST (MNCND[-])= 58.15 m, 95% CI: 56.25–60.04, HR: 5.92; **Figure 2F**).

## Discussion

The studies from the past 5 years have demonstrated that an increasing number of PTCs are being identified, which is based largely on an expansion in the use of diagnostic imaging and surveillance. Generally, most PTCs have an indolent course and an excellent outcome, with a 10-year RFS of 98% and 20-year cause-specific mortality rate of <1% (13, 14). PTCs arising in the thyroid isthmus have been reported in approximately 2.2–12.3% of all PTCs (4, 5). However, iPTCs may have a higher incidence of extrathyroidal invasion, CLNM, and multifocality than PTCs in the lateral lobes, because even a small tumor abuts the trachea and the thyroid capsule (15–18). J. Seok et al. observed that the presence of extrathyroidal extension of iPTC was 73.0%, which is significantly higher compared with PTCs in other sites (57.1%) (16). In the study of Song et al., the rate of CLNM of iPTCs was 71.1%, higher than that of other PTCs (40.3%) (18). Goldfarb et al. found that the proportion of multifocality was 48.6%, which was higher than in the lobes (39.8%) (6).

At present, there are no clear surgical guidelines for iPTCs, and the focus of debate is mainly on the extent of primary tumor resection. There are no specific guidelines for management of thyroid cancers confined to the thyroid isthmus. Several surgeons recommend that total thyroidectomy could be considered as an appropriate surgical treatment for PTC originating in the isthmus regardless of primary tumor size (6, 7). Only a few studies advocated isthmusectomy or wide field isthmusectomy as a suitable and reasonable surgical procedure for selected patients with small differentiated thyroid cancer limited to the isthmus without lymph node metastasis (8, 19, 20). Although the optimal extent of surgery for PTMC remains unclear, total thyroidectomy reduces tumor recurrence rates in patients with multifocal disease (21). In the current study, the rate of extrathyroidal extension and multifocality of iPTMC was 23.5% and 31.4%, respectively. Therefore, careful ultrasound evaluation should be performed in other sites of thyroid on iPTMCs preoperative for surgical decision-making (5).

The isthmus is located in front of the trachea and, due to its anatomical uniqueness, iPTCs may be detected early. Compared with PTC in other sites, iPTC tended to be smaller in size and had a higher proportion of iPTMC (22). iPTMCs accounted for 41.4–66.3% of all iPTCs (6, 23, 24), higher than that of other PTCs (35.7–48.8%) (25, 26). The ATA guidelines recommend fine-needle aspiration for nodules >5 mm in size when the patient falls within the high-risk category or if ultrasonographic examination shows manifestations that suggest malignancy (27). Previous studies have suggested that the recurrence rates and clinicopathologic features are similar in patients with PTMCs ≤5 mm and >5 mm in the lobes, except for lymph node metastasis and extrathyroidal extension (28, 29). However, the clinical significance and recurrence of iPTMCs ≤5 mm and >5 mm and their optimal management remain unclear. In our study, the rates of multifocality and CLNM were significantly lower in the ≤5 mm than in the >5 mm group. Female patients of iPTMC ≤5 mm were higher than that in the >5 mm. However, there were no statistically significant differences in extrathyroidal extension and TNM stage. Although male sex, lymph node metastasis, and multifocality are associated with poorer prognosis (30, 31), there were no significant differences in recurrence between the two groups. With a median follow-up of 49.5 months, the RFS of iPTMCs ≤5 mm and >5 mm did not have statistical differences. Time-dependent ROC analysis was conducted to clarify the cutoff point of tumor diameter in the current study. However, there were no statistically significant differences for recurrence.

On the study of Luo et al., tumor size of iPTC >11 mm was significantly associated with lymph node metastasis (23). Wang et al. found that tumor size >7 mm was associated with CLNM (32). Our findings revealed that the incidence of lymph node metastasis was significant high (52.1%) in iPTMC >5 mm group. However, the incidence of CLNM was relatively high in iPTMC >5 mm group when age <55 years, male sex, multiple tumor, and extrathyroidal extension existing showing no significant difference to that of same clinicopathologic factors in iPTMC ≤5 mm group. Therefore, we can infer that when the size of iPTMC is >5 mm, relatively high incidence of lymph node metastasis may occur when reaches certain risk factors. In fact, the correlation between lymph node metastasis and tumor recurrence was reported to be significant. CLNM was considered to be an aggressive clinical feature of PTMC related to higher incidence of recurrence (30, 31, 33). In the present study, the Kaplan–Meier survival analyses indicated that the RFS was better for iPTMCs without CLNM compared with that with CLNM. Of the nine cases of recurrence, one (11.1%) recurrence in a level VI lymph nodes and eight (88.9%) recurrences in lateral neck lymph nodes. Level III lymph nodes metastases accounted for 77.7% of all relapse cases.

With the comparison of clinicopathological variables of patients with and without recurrence, the relapse cases were found to be a higher CLNM (77.8%) and higher numbers of metastasis lymph nodes (*p* = 0.02). Several studies have found that the risk of recurrence was positively associated with a higher number of lymph nodes metastases at initial surgical operation (34, 35). The presence of >5 metastatic lymph nodes was associated with a significantly worse RFS than patients with <5 metastatic lymph nodes (36, 37). Interestingly, the present study shows that the presence of more than two metastatic lymph nodes is also associated with a significantly worse RFS. Using the existing data of iPTMC, we performed ROC curve analysis between the number of CLNM and recurrence. The AUC value was 0.729, cutoff value was 2.46, sensitivity was 55.6%, and specificity was 86.0%. The Kaplan–Meier survival analyses also indicated the significant differences of RFS between patients with ≥3 and <3 metastatic lymph nodes (*p* = 0.003). Furthermore, Cox proportional hazards model was used to identify risk factors for recurrence. On univariate analysis, risk factors for recurrence were extrathyroidal extension and ≥3 MNCND. Multivariate analysis indicated that the only independent risk factor for recurrence was ≥3 MNCND (HR, 4.566; 95% confidence interval, 1.18–17.66; *p* = 0.028). This showed that the model has a certain function in predicting recurrence and help clinicians for the decision of radioiodine administration and the modulation of follow-up modalities.

Our present study had several limitations. First, the sample size of the iPTMC was relatively small, which may cause a selection bias. Second, a relatively short follow-up time may be potential biases affecting the risk factor analyses and results. The retrospective nature of our study may also have led to selective bias. The larger number of patients with a longer period of follow-up is needed to overcome these limitations.

## Conclusions

In conclusion, our findings demonstrate that the recurrence rates are similar in patients with iPTMCs ≤5 mm and >5 mm. Nonetheless, iPTMCs >5 mm was more likely to be associated with pathological features such as multifocality and CLNM. Higher risk of CLNM should be considered in evaluation and surgical decision of iPTMC >5 mm when reaches some risk factors. The male gender, extrathyroidal extension, and CLNM were associated with recurrence of iPTMCs except for multifocality. The numbers of metastasis nodes for CND ≥3 may be an independent predictor for recurrence, which could help clinicians for the decision of radioiodine administration and the modulation of follow-up modalities.

## Data availability statement

The original contributions presented in the study are included in the article/supplementary material. Further inquiries can be directed to the corresponding author.

## Ethics statement

Written informed consent was obtained from the individual(s) for the publication of any potentially identifiable images or data included in this article.

## Author contributions

FZ, and YW conceived the idea and designed the research. XX and FL collected data and followed up patients. FZ, LZ and YS participated in statistical analysis and article writing. All authors contributed to the article and approved the submitted version.

## Funding

This work was supported by Zhejiang medical science and technology projects [2022KY786].

## Conflict of interest

The authors declare that the research was conducted in the absence of any commercial or financial relationships that could be construed as a potential conflict of interest.

## Publisher’s note

All claims expressed in this article are solely those of the authors and do not necessarily represent those of their affiliated organizations, or those of the publisher, the editors and the reviewers. Any product that may be evaluated in this article, or claim that may be made by its manufacturer, is not guaranteed or endorsed by the publisher.
